# Minimum Inhibitory Concentration Increase in *Clostridioides difficile* Isolates from Patients with Recurrence: Results from a Retrospective Single-Centre Cohort Study

**DOI:** 10.3390/microorganisms13071515

**Published:** 2025-06-28

**Authors:** Pietro Valsecchi, Erika Asperges, Marta Corbella, Greta Banfi, Marcello Maffezzoni, Nicolò Amarasinghe, Riccardo Drago, Flavia Virga, Filippo Costanzo, Francesca Calabretta, Paolo Sacchi, Patrizia Cambieri, Antonio Di Sabatino, Fausto Baldanti, Raffaele Bruno

**Affiliations:** 1 Infectious Diseases I Unit, IRCCS Policlinico San Matteo Foundation, 27100 Pavia, Italyraffaele.bruno@unipv.it (R.B.); 2 Clinical Microbiology and Virology Unit, IRCCS Policlinico San Matteo Foundation, 27100 Pavia, Italy; m.corbella@smatteo.pv.it (M.C.);; 3 Department of Clinical, Surgical, Diagnostic and Pediatric Sciences, University of Pavia, 27100 Pavia, Italy; greta.banfi01@universitadipavia.it (G.B.);; 4 Infectious and Tropical Diseases Unit, Department of Medicine and Surgery, University of Insubria-ASST-Sette Laghi, 21100 Varese, Italy; 5 Internal Medicine Unit, IRCCS Policlinico San Matteo Foundation, 27100 Pavia, Italy; 6 SSD Stewardship Antibiotica, ASST, 27100 Pavia, Italy; filippoccostanzo@gmail.com

**Keywords:** *Clostridioides difficile*, antimicrobial resistance, vancomycin, metronidazole, tigecycline, ciprofloxacin

## Abstract

Antimicrobial susceptibility testing (AST) is not routinely performed for C. difficile infection (CDI); however, reports of antimicrobial resistance to various antibiotics have increased. This study aimed to assess the rate of antimicrobial resistance to four antimicrobials (vancomycin, metronidazole, tigecycline, and ciprofloxacin) to assess risk factors for antimicrobial resistance and evaluate MIC variation in patients with recurrence. Data from consecutive patients with CDI admitted to our institution between 1 January 2022 and 30 April 2023 were collected. We performed AST with gradient diffusion and NAAT to evaluate the presumptive presence of R027/NAP1 and toxin production genes. Antimicrobial susceptibility testing was performed on 108 available isolates. We did not find any resistance to vancomycin (median MIC 0.5 μg/mL), metronidazole (median MIC 1 μg/mL), and tigecycline (median MIC 0.016 μg/mL), while resistance to ciprofloxacin was detected in all the samples. Among the recurrent isolates, 37.5% displayed a 2-fold MIC increase for vancomycin, 75% for metronidazole, and 37.5% for tigecycline. After stratifying clinical outcomes according to vancomycin MIC, patients with higher MIC experienced increased 28-day mortality (*p* value 0.009). Our results were concordant with European surveillance data. MIC increase in all tested antibiotics in patients with CDI warrants further research since decreased susceptibility has been associated with clinical failure.

## 1. Introduction

*Clostridioides difficile* is a Gram-positive, anaerobic, spore-forming bacterium. Disruptions to gut microbiota, such as those due to antibiotic use or proton pump inhibitors, can promote *C. difficile* colonisation and the proliferation of its vegetative cells. Colonisation is not necessarily pathogenic, but it can be persistent due to endospores and gut adhesions, and it is the first step to symptom development [1].

Symptoms of *C. difficile* infection (CDI) are due to toxin A (TcdA) and toxin B (TcdB) in the chromosomal pathogenicity locus (PaLoc) of toxigenic strains. TcdA and TcdB are glucosyltransferases that inhibit Ras-related glucosyltransferase (GTP)-binding proteins, which regulate the actin cytoskeleton organisation. A third toxin, CDT (binary toxin), is produced by some strains, including hypervirulent ribotypes (RT027 and RT078). The toxin damages intestinal cell integrity, enhances bacterial adherence, and further increases disease severity [2].

CDI includes a broad range of clinical manifestations, ranging from mild or moderate diarrhoea, pseudomembranous colitis, to life-threatening manifestations such as toxic megacolon and bowel perforation [3].

A 2016–2017 report by the European Centre for Disease Control and Prevention showed a *Clostridioides difficile* infection (CDI) incidence of 3.48 cases per 10,000 patient-days, which was higher in tertiary hospitals. The 1-year attributable mortality due to CDI is around 7.9%, but the main concern is the high risk of recurrence that leads to further hospitalisation. This risk is up to 20–25% after the first CDI episode and increases with each episode, reaching more than 60–65% after three episodes [3,4].

Other risk factors for recurrence include older age, multiple comorbidities, prior hospitalisation, healthcare-associated CDI (HA-CDI), and the use of proton pump inhibitors, especially if started during a previous episode of CDI [3,5].

First-line therapy for CDI, according to guidelines from the European Society on Microbiology and Infectious Disease (ESCMID) and the Infectious Disease Society of America (IDSA), is fidaxomicin, with oral vancomycin as an alternative. Despite a decrease in vancomycin treatment success in recent years, this antibiotic retains high cure rates for CDI. Therefore, it represents a valuable alternative to treat patients with a first episode of CDI and may be administered using a tapered and pulsed scheme to prevent recurrence [6,7]. Furthermore, vancomycin has the advantage of lower acquisition costs compared to fidaxomicin [8]. Since studies evaluating the cost-effectiveness of the recommended regimen are limited, ESCMID guidelines suggest using a risk stratification method to select patients who will benefit more from fidaxomicin treatment in settings where access to this drug is limited [7].

New treatment options available to prevent recurrence after previous episodes of CDI are monoclonal antibodies directed toward toxin B (bezlotoxumab) and faecal microbiota transplantation [6,7].

The diagnosis of CDI is based on glutamate dehydrogenase (GDH) antigen detection, the Nucleic Acid Amplification Test (NAAT), and detection of toxin A and B via enzyme immunoassay (EIA) rather than microbiological culture. Therefore, antimicrobial susceptibility testing (AST) is not routinely performed [9].

Nevertheless, AST performed for surveillance purposes has shown an increase in resistance rates in recent years. Incidence of vancomycin and metronidazole resistance in *C. difficile* varies according to different settings and methods, but a recent meta-analysis showed a weighted pooled antimicrobial resistance (WPR) of 3% for vancomycin and 5% for metronidazole [10,11]. Several resistance mechanisms have been described in *C. difficile*. These include the resistance-associated genes harboured in the bacterial chromosome, mobile genetic elements (MGEs), alterations in the antibiotic targets of antibiotics and/or in metabolic pathways, and biofilm formation [12,13,14]. Current data suggest that metronidazole resistance is due to several alterations in several metabolic pathways, involving the activity of nitro reductases, iron uptake, and DNA repair, but can also be associated with the acquisition of pCD-METRO plasmid (7-kb) [12]. Vancomycin resistance might be due to amino acid changes in peptidoglycan biosynthesis-associated proteins such as MurG., alterations in binding sites, VanSR-controlled expression of different van gene clusters, acquisition of plasmids such as pX18–498 from other bacteria, efflux pumps, and biofilms [13]. Notably, a vanGcd cryptic gene cluster homologous to the enterococcal vanG cluster was found in *C. difficile*, and mutations in the gene can confer resistance to the agent [14].

Resistance to fidaxomicin is anecdotal and has been linked to mutations in the RNA polymerase b subunit, such as Val1143Gly and Val1143Asp. However, carrying these mutations causes a reduction in bacterial fitness, and it is associated with defects in growth, sporulation, and toxin production [15]. Although fluoroquinolones, like ciprofloxacin, are not recommended for CDI, their resistance seems to be a hallmark of hypervirulent strains and has been associated with higher mortality. It is mediated by amino acidic substitutions harboured in two DNA gyrase subunits, GyrA and/or GyrB, acquired when the environmental concentration of fluoroquinolones is not able to inhibit *C. difficile* [11,13].

Tigecycline is an antimicrobial of the tetracycline class and has in vitro activity against *C. difficile*; it has been recently suggested as a possible adjunctive treatment for severe CDI, based on retrospective observational studies [7]. Resistance to tetracycline in *C. difficile* is thought to be associated with transposons Tn5397, Tn916, or Tn916-like family, and Tn6164. While tetracycline resistance is increasing in *C. difficile* isolates, tigecycline retains low MIC values for this organism [13].

Factors associated with reduced susceptibility to antimicrobials have been poorly described; however, the presence of resistance has been frequently linked to ribotype 027 and toxin B production [16,17].

R027 strains tend to be associated with increased vancomycin resistance due to frequent association with a Thr115Ala mutation in the regulatory gene VanR that results in constitutive expression of vanG, leading to decreased vancomycin affinity (MIC50 and MIC90 values of 2 and 4 µg/mL) [18,19].

The clinical impact of antimicrobial resistance in CDI remains uncertain, as the high concentrations of vancomycin and fidaxomicin in the stool may offset the effects of resistance. However, metronidazole resistance has been associated with treatment failure, likely due to lower stool drug concentrations [20].

The latest Italian survey on *C. difficile* AMR was performed by the Italian Istituto Superiore di Sanità (ISS) between 2006 and 2016. There was no vancomycin resistance reported, and only one case of a human isolate with metronidazole resistance was recorded. IRCCS Fondazione Policlinico San Matteo is a tertiary care hospital located in the Lombardia region. The survey results are likely representative of the local epidemiology since this region accounted for 32% of the total sample [21].

Considering the epidemiological changes reported worldwide, this study’s primary aim was to assess the prevalence of *C. difficile* resistance to vancomycin, metronidazole, ciprofloxacin, and tigecycline. Secondary objectives include evaluating risk factors for resistance, MIC variations in patients with recurrence, and clinical outcomes according to MIC values. The study population was adult patients admitted with CDI.

## 2. Materials and Methods

### 2.1. Study Procedures

This is a retrospective single-centre, cohort observational study.

The study population included patients aged 18 years or more admitted to IRCCS Fondazione Policlinico San Matteo with a microbiologically confirmed diagnosis of CDI between 1 January 2022 and 30 April 2023.

The study period was chosen considering the incidence of CDI in the centre and to ensure an adequate sample size for the primary objective of this study.

This study was approved by the Fondazione IRCCS Policlinico S. Matteo Internal Review Board (protocol number 39948/2023). Informed Consent was waived by the Fondazione IRCCS Policlinico S. Matteo Internal Review Board due to the retrospective nature of this study.

Stool samples of patients admitted to Fondazione IRCCS Policlinico San Matteo with clinical suspicion of CDI were processed by the Microbiology laboratory.

An episode of CDI was defined as clinical findings compatible with CDI and microbiological evidence of *C. difficile* free toxins by EIA without reasonable evidence of another cause of diarrhoea or a clinical picture compatible with CDI and a positive NAAT, or positive toxigenic *C. difficile* culture or pseudomembranous colitis as diagnosed during endoscopy, after colectomy, or on autopsy, in combination with a positive test for the presence of toxigenic *C. difficile*. Diarrhoea was defined as ≥3 loose stools in 24 h. Local protocols required a liquid stool sample consistency for testing (Bristol scale 5–7) and no administration of laxatives within 48 h of testing. Analyses were performed on fresh samples to avoid toxin degradation. Patients with positive microbiological findings for *C. difficile* without clinical findings compatible with CDI were excluded. Recurrence was defined as the reappearance of symptoms within 8 weeks after a previous CDI episode, provided the symptoms of the prior episode had resolved after completion of initial treatment [4].

For each included patient, clinical data were retrieved from medical charts and electronic records for each patient. Collected data included epidemiological data and risk factors (comorbidities, use of proton pump inhibitors, use of antibiotics, and previous use of anti-*C. difficile* therapy).

A case was defined as community-associated if the *C. difficile*-positive stool specimen was collected on an outpatient basis or within 3 days after hospital admission in a person with no documented overnight stay in a healthcare facility in the preceding 12 weeks [6].

Severe CDI was defined as an episode of CDI with a white blood cell count of >15,109 cells/µL or a serum creatinine level > 1.5 mg/dL > or an increase of 50% from the baseline [22].

*C. difficile*-positive stool samples were subjected to alcohol shock by preparing a 1:1 suspension of faecal sample and 95% ethanol according to ECDC standard procedures [9], followed by vortexing and incubation at room temperature for 1 h. Subsequently, 50–100 microliters of the suspension was inoculated onto Schaedler Agar + 5% sheep blood (Biomérieux, Marcy L’Etoile, France) and incubated at 37 °C under anaerobic conditions for 3 days. AST was performed on all available isolates using gradient diffusion (MIC Test Strip, Liofilchem srl, Roseto degli Abruzzi (TE), Italy) and read by two independent microbiologists. The results were interpreted according to the European Committee on Antimicrobial Susceptibility Testing (EUCAST) reference breakpoints for vancomycin and metronidazole and according to the Clinical Laboratory Standard Institute (CLSI) for tigecycline and ciprofloxacin [23,24]. Antimicrobials tested were selected due to their widespread use for the treatment of CDI and increased reports of antimicrobial resistance (vancomycin and metronidazole) due to their therapeutic potential (tigecycline) or epidemiological link with *C. difficile* virulence (ciprofloxacin). According to previous reports, gradient diffusion may result in the underestimation of MICs [11], which is why the agar dilution test is the reference for *C. difficile* antimicrobial susceptibility testing. Nevertheless, we performed gradient diffusion because it allows testing of multiple antimicrobials and has shown good concordance with agar dilution [25].

NAAT (GeneXpert, Cepheid, Sunnyvale, CA, USA) was used to identify presumptive NAP01/R027 (due to tcdCΔ117 deletion) and toxin production genes. This assay is characterised by a sensitivity of 100% and a specificity of 97% in detecting tcdCΔ117 deletion [26]. No further molecular testing or sequencing was performed.

### 2.2. Statistics

The sample size was calculated on the primary endpoint of vancomycin and/or metronidazole resistance prevalence. The statistical unit was the positive stool sample corresponding to an infectious episode. We hypothesised a prevalence of 5% according to the results of a recent meta-analysis [10]. Therefore, this study would require a sample size of 130 for estimating the expected proportion with 5% absolute precision and 99% confidence [27].

All analyses were performed using RStudio version 4.4.0 [28]. We described continuous variables with the mean and standard deviation or the median and 25th–75th percentiles for skewed distributions; we described categorical data as counts and percentages. We compared characteristics between groups with the Student *t* test or the Kruskal–Wallis test and the Fisher exact test, respectively. A 2-sided *p*-value of 0.05 was considered statistically significant. Prevalence of antimicrobial resistance was calculated by dividing the number of resistant isolates by the total number of samples tested.

Clinical outcomes after stratifying for MIC values were compared using the Fisher exact test: a 2-sided *p*-value of 0.05 was considered statistically significant.

## 3. Results

### 3.1. Patients

We enrolled 108 patients; 46% of them were males and 54% were females. Median age was 76 years (interquartile range (IQR) 62–84). Demographic and clinical characteristics are presented in Table 1: the median Charlson Comorbidity Index (CCI) was 5 (IQR: 4–7); the most prevalent comorbidities were cardiovascular disease, COPD, and solid tumours.

Eighty-six percent of the patients presented with healthcare-acquired CDI, and 47% presented with severe clinical presentation.

For 78% of the patients, it was the first episode, for 16% it was the first recurrence, and 6% had two or more recurrences. Most patients presented well-known risk factors for CDI since nearly 90% received antimicrobial treatment in the three months preceding the infection, and nearly 70% were receiving PPI. Almost 20% of patients had already been treated with vancomycin for a previous CDI episode. For the current episode, the preferred treatment was vancomycin in 74.7% of patients, followed by the combination of vancomycin and metronidazole in 17.7%.

NAAT identified presumptive NAP1/R027 in 30% of patients, toxin B production in 83% of patients, and binary toxin production in 42% of patients.

With regards to clinical outcomes, 16.9% of patients experienced a subsequent recurrence within 8 weeks of the index episode, and 7.3% of them died within 28 days.

### 3.2. MICs of Vancomycin, Metronidazole, Tigecycline, and Ciprofloxacin

**Figure 1:**
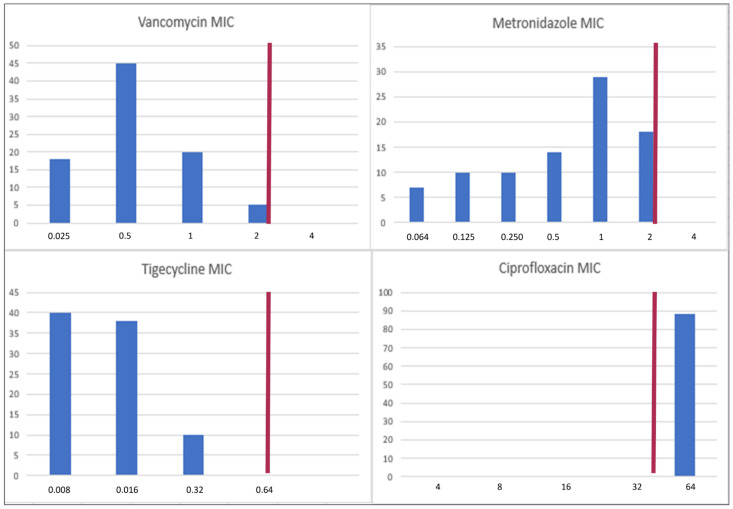
MIC distribution of the isolates. The red line represents the breakpoint.

All 108 isolates were available for antimicrobial susceptibility testing. The MIC distribution is represented in Figure 1. All the isolates were resistant to ciprofloxacin but sensitive to the other three antibiotics, although MIC distribution varied. The median MICs for vancomycin, metronidazole, tigecycline, and ciprofloxacin were 0.5 μg/mL, 1 μg/mL, 0.016 μg/mL, and 32 μg/mL.

### 3.3. MIC Variation in Subsequent Episodes

**Figure 2:**
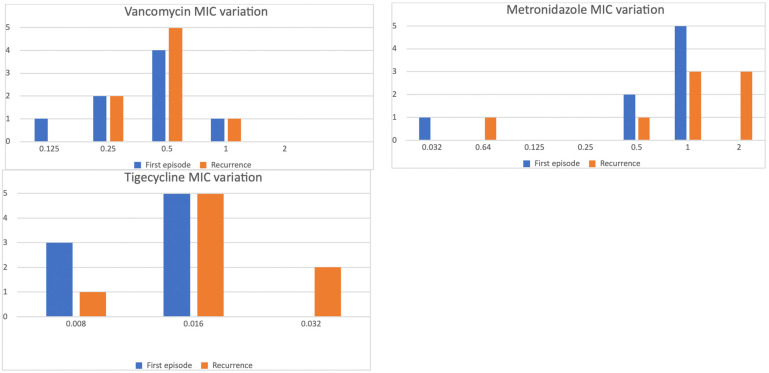
MIC distribution of the isolates in the first episode (blue) and the recurrence (orange), showing a trend toward MIC increase.

Sixteen isolates from both the first episode and recurrence were available for eight patients: three (37.5%) displayed a twofold MIC increase for vancomycin, six (75%) a twofold increase for metronidazole, and three (37.5%) a twofold increase for tigecycline. The variation in MIC distributions is presented in Figure 2. The observed increase in MIC did not result in a shift across the susceptibility breakpoints.

### 3.4. Variables Associated with MIC Distributions

We did not find any significant correlation between the antibiotics’ MICs and possible risk factors: episode number, previous vancomycin administration, and mode of acquisition (healthcare versus community). Similarly, we did not find any statistically significant MIC according to the presence of presumptive NAP1/027 or toxin production genes (Supplementary Table S1, Supplementary Figure S1).

### 3.5. Relationship of Vancomycin, Metronidazole, and Tigecycline MICs with Clinical Outcomes

While the proportion of recurrence was similar after stratifying patients according to vancomycin MIC values, 28-day mortality occurred more frequently in patients with an MIC of 2 μg/mL compared to other groups, as shown in Table 2. This difference was statistically significant using the Fisher exact test (*p* value 0.009). The proportion of recurrence and 28-day mortality was not significantly different after stratifying patients according to metronidazole and tigecycline MIC.

## 4. Discussion

The present study aimed to evaluate the rate of antimicrobial resistance of *C. difficile* isolates recovered from patients admitted with CDI in our institution and to evaluate the risk factors associated with decreased antimicrobial susceptibility.

The included population was consistent with the epidemiology of CDI: patients were old, with multiple comorbidities, and frequently received treatment with PPIs or antimicrobials in the three months preceding CDI infection [4,5].

The vast majority of patients presented with healthcare-acquired CDI, with a high proportion of presumptive R027 strains.

We did not find any isolates resistant to vancomycin, metronidazole, and tigecycline, while all the isolates were resistant to ciprofloxacin.

While worldwide weighted pool resistance to vancomycin and metronidazole was found to be 3% and 5% [10], their frequency is variable according to different world regions, reaching up to 34% in a cohort of patients with CDI in the US [29]. Our results are consistent with those reported in Southern and Western Europe, while increased metronidazole resistance has been described in Eastern Europe [30,31]. It is worth noting that metronidazole MICs in isolates from our cohort were close to the breakpoint and frequently higher than the geometric mean metronidazole MIC from a recent collection of European isolates (0.29 mg/L). Similarly, MIC distribution for metronidazole showed a shift closer to the breakpoint value when compared to the latest Italian survey performed between 2006 and 2016 [21]. The high proportion of presumptive NAP01/R027 in our cohort can explain this finding, as this ribotype was associated with a higher geometric mean metronidazole MIC (1.87 mg/L) [31].

Interestingly, all the isolates demonstrated low MICs for tigecycline, which has been recently introduced in the ESCMID guidelines as a possible therapeutic option in severe CDI due to favourable outcomes in a retrospective cohort study [7,32].

Universal ciprofloxacin resistance confirms trends observed in Italy and Europe during the last decades [33,34]. Fluoroquinolone resistance is mediated by mutation in the *gyr* genes, encoding DNA gyrase subunits, and the results from subinhibitory concentrations of this antimicrobial class due to their widespread use in the healthcare setting and the community. Furthermore, fluoroquinolone exposure is a well-established risk factor for CDI, and ciprofloxacin upregulates the expression of both TcdA and TcdB in hypervirulent strains of *C. difficile* [35,36].

A key finding of our study was the frequent increase in MICs observed when comparing isolates from initial episodes to those from subsequent recurrences in the same patients. This pattern affected all tested antibiotics, even those not used in treating the index infection. Retrospective studies have previously described vancomycin and metronidazole MIC increases in patients with recurrence. This phenomenon was associated with hypervirulent strains and binary toxin production [17,37]. A phase 2 clinical trial evaluating the efficacy of rinidazole compared to vancomycin described MIC increases for vancomycin and metronidazole of similar magnitude in a small sample of recurrent CDIs [38].

When comparing these findings with the aforementioned studies, it is worth noting that we described a frequent increase in tigecycline MIC, despite no patient receiving this drug. Albeit interesting, this finding needs to be confirmed in larger cohorts since only a small subset of isolates from recurrences were available for AST. Due to this limited sample, these results are mainly descriptive. Therefore, it is not possible to derive strong conclusions, but we can only hypothesise the underlying mechanisms. The observed MIC increases may be related to biofilm formation, which is a common phenomenon in *C. difficile* isolates previously linked to reduced susceptibility to vancomycin and metronidazole [11,13,39]. Biofilms may facilitate the selection of more tolerant strains after initial treatment, potentially contributing to recurrent infections. Although we did not directly evaluate biofilm production in our isolates, this mechanism could explain our observations.

Several bacterial factors, such as ribotype 027, binary toxin, and toxin B production, have reduced antimicrobial susceptibility to both metronidazole and vancomycin [18].

R027 strains tend to be associated with increased vancomycin resistance due to frequent association with a Thr115Ala mutation in the regulatory gene VanR that results in constitutive expression of vanG, leading to decreased vancomycin affinity (MIC50 and MIC90 values of 2 and 4 µg/mL) [18,19].

When stratifying MIC values according to virulence genes, we did not find any significant differences associated with their expression. All the isolates in our cohort were susceptible to vancomycin; therefore, R027 strains were probably harbouring wild-type Thr115, which is characterised by MIC50 and MIC90 of 0.5 and 2 μg/mL, respectively [19].

Therefore, the impact of virulence genes on antimicrobial susceptibility is likely less relevant when assessing values below the breakpoint.

Despite the difficulties of assessing the possible clinical impact of MIC variation within the breakpoint value, we evaluated clinical outcomes stratified by MIC values.

Isolates with higher MICs were associated with increased mortality, despite similar recurrence rates. This finding should be interpreted cautiously, since mortality in CDI is associated with several factors, like age, disease severity, and comorbidities. The overall 28-day mortality in our cohort was 7.3%, which is similar to previous epidemiological data on CDI [3]. The low number of events made it impossible to adjust for these concurrent factors.

Despite all these limitations, we can speculate that isolates with MIC values at the upper end of the susceptibility range could reduce the effectiveness of vancomycin treatment. In patients with severe disease or at increased risk of mortality due to the underlying comorbidities, higher MICs could negatively influence clinical outcomes.

The principal strength of this study is that it shows MIC increase in isolates from recurrence in a subset (albeit limited) of real-life patients with CDI.

Several limitations of this study must be acknowledged. Firstly, the retrospective and single-centre design makes these findings poorly generalisable to other settings and implies the presence of missing data.

Secondly, we did not perform susceptibility testing for fidaxomicin, which represents the recommended treatment for CDI. The reports of fidaxomicin resistance have been anecdotal and associated with fitness costs for *C. difficile*; therefore, its impact seems limited [15].

Additionally, our molecular testing detected only certain genes rather than providing comprehensive genotyping or ribotyping [16,17]. Finally, the final sample was slightly underpowered compared to the planned size.

Antimicrobial resistance has become more frequent in CDI, but its influence on clinical outcomes is debated. Traditionally, the high intestinal concentrations of vancomycin achieved during therapy (exceeding MICs by 10–100 times) were thought to overcome modest increases in resistance [20].

This assumption has been recently questioned by a retrospective cohort study by Eubank et al. They found that decreased susceptibility was associated with lower sustained clinical cure, with the effect driven primarily by antibiotic failure rather than increased recurrence rates [29].

With this in mind, our results have potential clinical implications. Reduced antimicrobial susceptibility in isolates from recurrences could potentially lead to lower treatment effectiveness. While we could not confirm this hypothesis due to the absence of antimicrobial resistance and the small sample size of our cohort, this could have a relevant impact in settings with high rates of vancomycin and metronidazole resistance.

Interestingly, we found an increased mortality in patients with higher MICs. Despite all the limitations described above, these findings confirm the possible role of antimicrobial susceptibility in clinical outcomes, while microbiota preservation or restoration is crucial to prevent recurrence. With this in mind, antimicrobial susceptibility testing could become an indicator to guide therapy in CDI, particularly in patients with severe disease or those not responding to antimicrobial treatment.

## 5. Conclusions

The results of this study provide important insights for both antimicrobial resistance surveillance and clinical management of CDI. We did not find resistance to commonly used antibiotics (vancomycin, metronidazole, and tigecycline), but we observed MIC increases in recurrent infections, a fact which warrants close attention.

Due to the retrospective and single-centre nature of this study, our results are hypothesis-generating rather than hypothesis-testing. Future perspectives include evaluating MIC variations in recurrent isolates in larger prospective and possibly multicentric cohorts to confirm this finding and test possible underlying mechanisms.

When evaluating antimicrobial susceptibility differences in recurrent isolates, future studies should focus on a mechanistic explanation through genome sequencing and evaluation of resistance genes. We hypothesise that biofilm formation can partially explain our findings; therefore, evaluating biofilm formation in recurrent isolates could clarify its role in the development of antimicrobial resistance. Investigating the role of biofilm in CDI recurrence could open the path for the development of new therapeutic strategies.

In light of the recent paradigm shift in the understanding of clinical implications of antimicrobial resistance in CDI infections, studies on the real-life impact of MIC increase on patients with recurrences are warranted.

## Figures and Tables

**Table 1:** Patients’ characteristics. The results are presented with counts and percentages for categorical variables, while continuous variables are presented as the median and interquartile range. Percentages are based on available data for each variable. CDI: *Clostridioides difficile* infection.

Patients (Total)	108
Epidemiology	
Age [years]	76 (62–84)
Males/females	50/58 (46.0%/54.0%)
Charlson Comorbidity Index	5 (4–7)
Cardiovascular disease	32 (37.0%)
Peripheral venous disease	12 (13.8%)
Stroke	12 (13.8%)
Chronic Obstructive Pulmonary Disease	21 (24.1%)
Liver disease	13 (14.9%)
Solid malignancy	16 (18.4%)
Haematological malignancy	2 (2.4%)
Previous hospitalization	38 (44.2%)
Current episode of CDI	
Hospital/community-acquired CDI	74/12 (86.0%/14.0%)
Severe CDI	39 (47.0%)
Episode: first/second/third or more	67/14/5 (77.9%/16.3%/5.8%)
Duration of therapy [days]	10 (10–13.5)
NAP1/R027	30 (30.0%)
Binary toxin	42 (42.0%)
TcdB+	83 (83.0%)
ICU admission	2 (2.4%)
Recurrence	14 (16.9%)
Length of stay [days]	26 (15–42%)
28-day mortality	6 (7.3%)
Therapies	
Use of proton pump inhibitors before CDI	58 (69.1%)
Proton pump inhibitors started during CDI	7 (8.4%)
Recent use of antibiotics	76 (89.4%)
Previous use of vancomycin	17 (19.8%)
Previous use of metronidazole	7 (8.4%)
Previous use of fidaxomicin	3 (3.5%)
Treatment with vancomycin	59 (74.7%)
Treatment with fidaxomicin	5 (6.3%)
Treatment with metronidazole	1 (1.3%)
Treatment with vancomycin and metronidazole	14 (17.7%)

**Table 2:** Recurrence and 28-day mortality according to vancomycin, metronidazole, and tigecycline MICs. Outcomes are presented as counts and percentages; *p*-values refer to the Fisher exact test.

Outcome	Vancomycin MIC (μg/mL)					
	0.25	0.5	1	2		*p*
Recurrence	3/14 (21.4%)	6/35 (17.1%)	4/17 (23.5%)	1/5 (20%)		0.9
28-day mortality	1/14 (7.1%)	0/35 (0%)	2/17 (11.8%)	2/5 (40%)		**0.009**
	**Metronidazole MIC (μg/mL)**					
	**0.064**	**0.125**	**0.250**	**0.5**	**1**	
Recurrence	2/6 (33.3%)	1/7 (14.3%)	6/9 (11.1%)	3/10 (30%)	6/25 (24%)	0.8
28-day mortality	0/6 (0%)	0/7 (0%)	3/9 (33.3%)	0/10 (0%)	2/25 (8%)	0.13
	**Tigecycline MIC (μg/mL)**					
	**0.008**	**0.016**	**0.032**	**0.064**		
Recurrence	6/35 (17.1%)	8/29 (27.6%)	0/7 (0%)	-		0.3
28-day mortality	3/35 (8.6%)	1/29 (3.6%)	1/7 (14.3%)	-		0.38

## Data Availability

The data presented in this study are available on request from the corresponding author. The data are not publicly available due to privacy.

## Supplementary Materials

The following supporting information can be downloaded at https://www.mdpi.com/article/10.3390/microorganisms13071515/s1: Supplementary Table S1. Kruskal–Wallis test to compare MIC values and possible risk factors. Supplementary Figure S1. Violin plots of the MIC distribution for vancomycin, metronidazole, and tigecycline in relation to the presence of the NAP1/027 strain, binary toxin, and toxin b. The black square is set at the median value.
