# Supplementary material for: Minimum Inhibitory Concentration Increase in Clostridioides difficile Isolates from Patients with Recurrence: Results from a Retrospective Single-Centre Cohort Study
Source: Microorganisms. 2025 Jun 28;13(7):1515. doi: 10.3390/microorganisms13071515 (PMC12300278; doi:10.3390/microorganisms13071515)

Supplementary Table S1. Kruskal-Wallis test to compare MIC values and possible risk factors.

	Risk factor	MIC (median)	Interquartile range (25°-75°)	Chi-squared	p-value
Vancomycin	Binary toxin: present	0.50	0.50-0.50	0.53	0.47
	Binary toxin: absent	0.50	0.50-1.00		
	First episode	0.50	0.50-1.00	3.97	0.14
	Episode: first recurrence	0.25	0.25-0.25		
	Recurrence ≥2	1.00	1.00-1.00		
	Community acquired	0.25	0.25-0.50	1.59	0.21
	Hospital acquired	0.50	0.50-1.00		
	Previous use of vancomycin	1.00	0.50-1.00	0.40	0.53
	No previous use of vancomycin	0.50	0.50-0.50		
	NAP1/027: present	0.50	0.25-0.50	1.17	0.28
	NAP1/027: absent	0.50	0.50-1.00		
	Toxin B: present	0.50	0.50-1.00	0.11	0.74
	Toxin B: absent	0.50	0.50-1.00		
Metronidazole	Binary toxin: present	1.00	0.50-1.00	0.33	0.56
	Binary toxin: absent	0.50	0.25-1.00		
	Episode: first	1.00	0.50-1.00	2.323	0.31
	Episode: first recurrence	1.00	1.00-1.00		
	Recurrence ≥2	0.125	0.125-1.00		
	Community acquired	1.00	1.00-1.00	0.50	0.48
	Hospital acquired	1.00	0.50-1.00		
	NAP1/027: present	1.00	1.00-1.00	0.53	0.48
	NAP1/027: absent	0.50	0.50-1.00		
	Toxin B: present	1.00	0.50-1.00	0.70	0.40

Supplementary Table S1. Kruskal-Wallis test to compare MIC values and possible risk factors.

	Toxin B: absent	1.00	0.50-2.00		
Tigecyline	Binary toxin: present	0.016	0.008-0.016	2.47	0.12
	Binary toxin: absent	0.008	0.008-0.016		
	Episode: first	0.016	0.008-0.016	1.61	0.45
	Episode: first recurrence	0.016	0.016-0.016		
	Recurrence ≥2	0.008	0.008-0.008		
	Community acquired	0.008	0.008-0.016	0.15	0.70
	Hospital acquired	0.016	0.008-0.016		
	NAP1/027: present	0.016	0.008-0.016	0.05	0.83
	NAP1/027: absent	0.016	0.008-0.016		
	Toxin B: present	0.016	0.008-0.016	0.14	0.70
	Toxin B: absent	0.016	0.008-0.016		

Supplementary Figure S1. Violin plots of the MICs distribution for vancomycin, metronidazole and tigecycline in relation to the presence of strain NAP1/027 strain, binary toxin and toxin b. The black square is set at the median value.

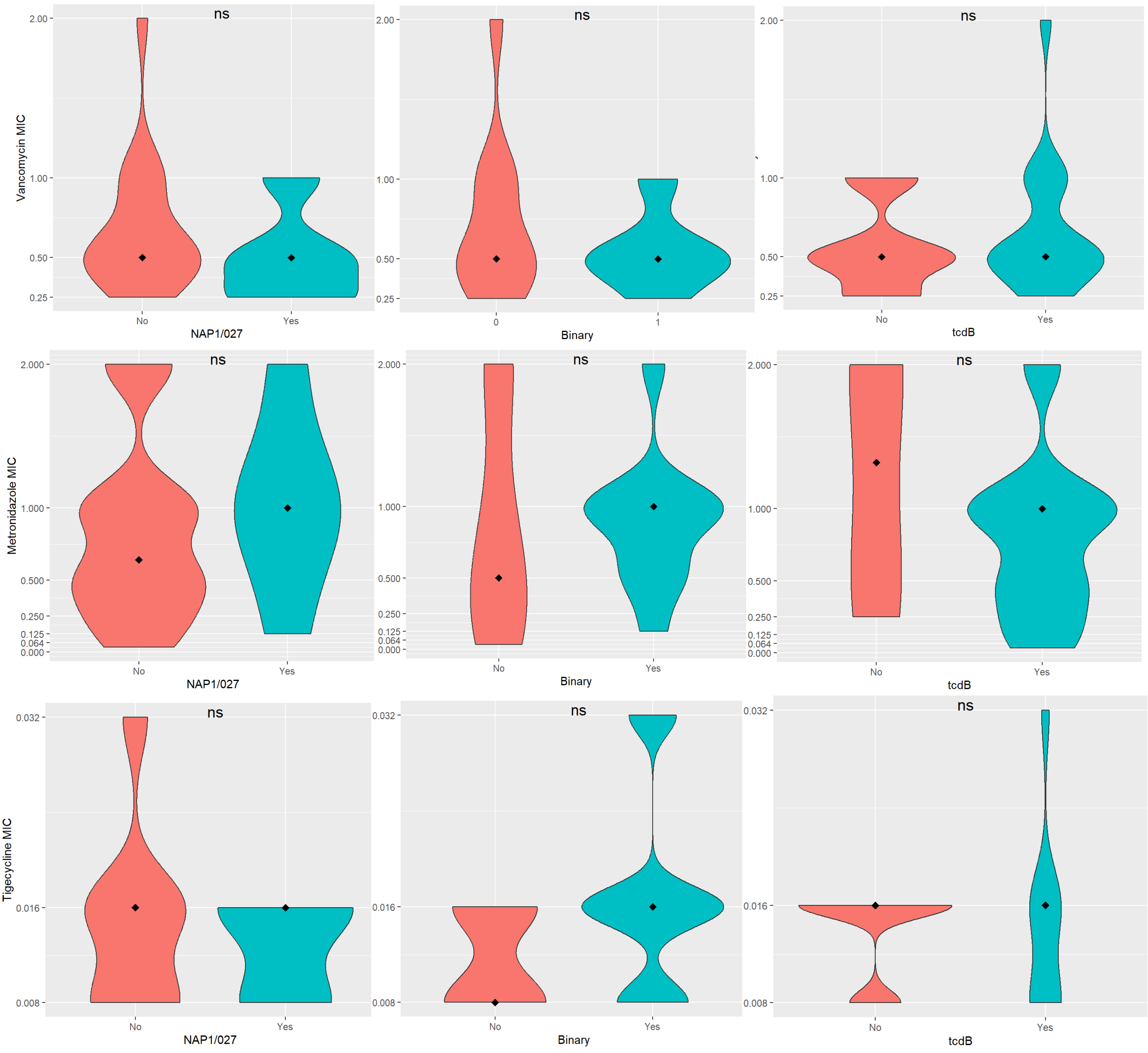

Supplement: Supplementary file 1 [file microorganisms-13-01515-s001.zip › microorganisms-3683096-supplementary.pdf]
